# Supplementary material for: Integrated 2D multi-fin field-effect transistors
Source: Nat Commun. 2024 Apr 29;15:3622. doi: 10.1038/s41467-024-47974-2 (PMC11058203; doi:10.1038/s41467-024-47974-2)
Supplement: Supplementary file 1 — Supplementary Information [file 41467_2024_47974_MOESM1_ESM.pdf]

Supplementary Information for

Integrated 2D multi-fin field-effect transistors

Mengshi Yu†, Congwei Tan†, Yuling Yin†, Junchuan Tang, Xiaoyin Gao, Hongtao Liu, Feng Ding*, Hailin Peng*

Corresponding to: hlpeng@pku.edu.cn; f.ding@siat.ac.cn

Supplementary Figures

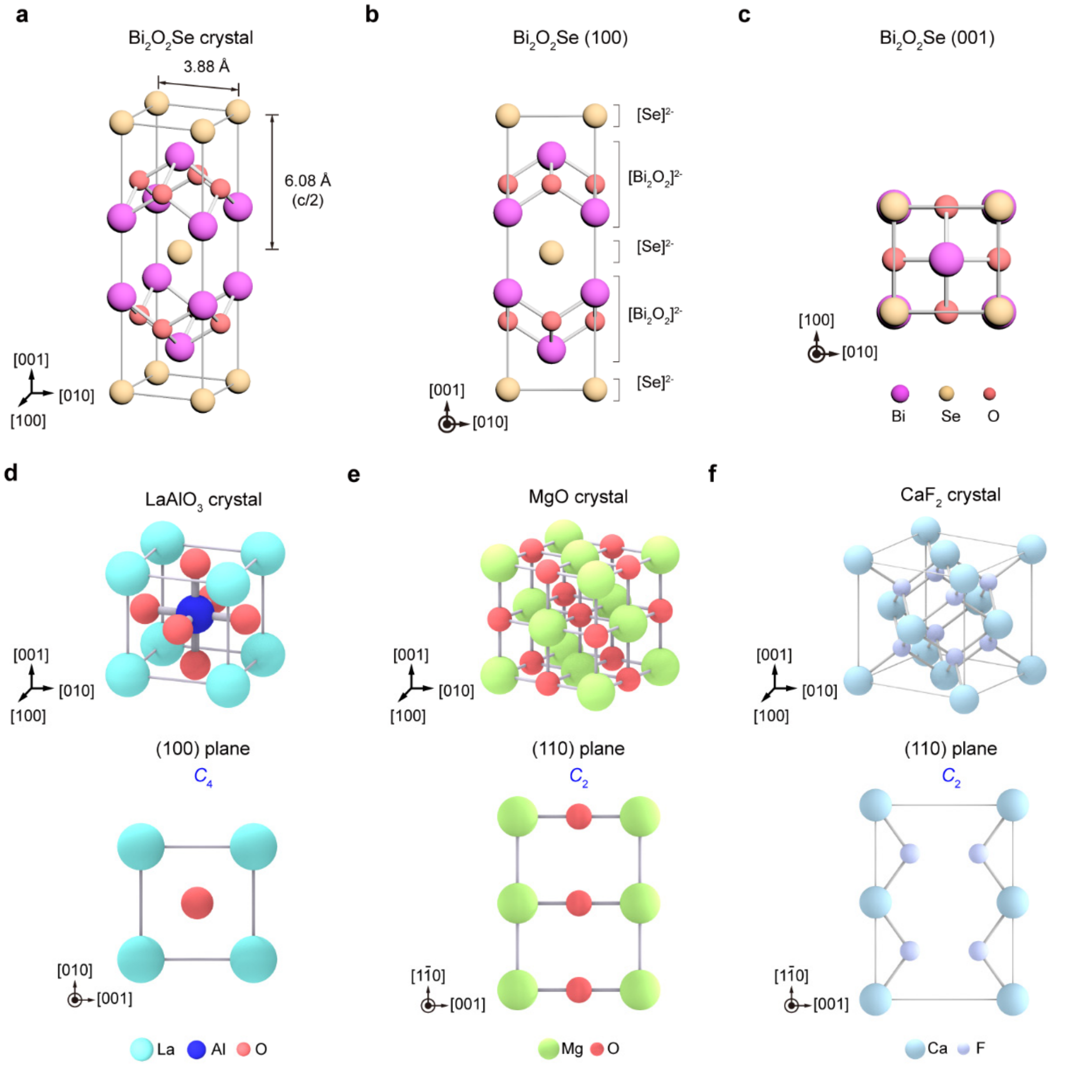

Supplementary Fig. 1. Schematics of crystal structure of 2D Bi_2O_2Se semiconductor and insulating substrates. a, 3D crystal structure of Bi_2O_2Se. **b,** Lateral view of Bi_2O_2Se crystal structure. **c,** top view of Bi_2O_2Se crystal structure. **d,** 3D structure and (100) facet lattice of $LaAlO_3$ crystal. **e,** 3D structure and (110) facet lattice of MgO crystal. **f,** 3D structure and (110) facet lattice of CaF_2 crystal.

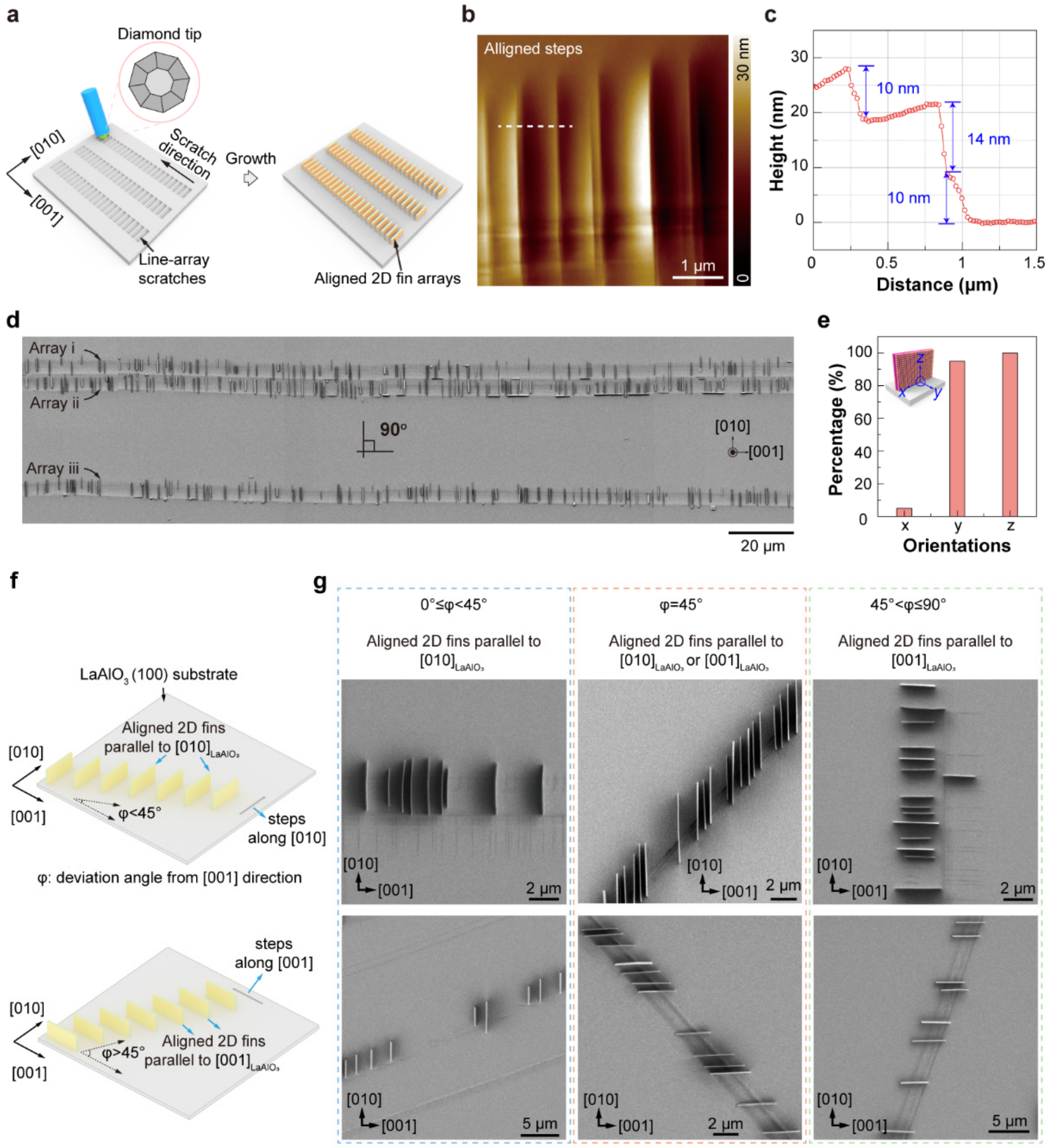

Supplementary Fig. 2. Creating process of artificially parallel steps using a diamond scraper. a, Schematic illustration of creating line-array scratches by diamond scraper. **b**, **c**, AFM image (**b**) and corresponding step height (**c**) of the artificial steps. **d**, SEM image of guided-grown 2D fin arrays while using a micromachined arm and a diamond scrape to control the step spacing. **e**, Statistical orientations of the 2D fin arrays in (**d**). **f,** Schematic illustration of different step orientations under different scratch directions. **g**, SEM images of 2D fin arrays with varying orientations resulting from scratches in different directions.

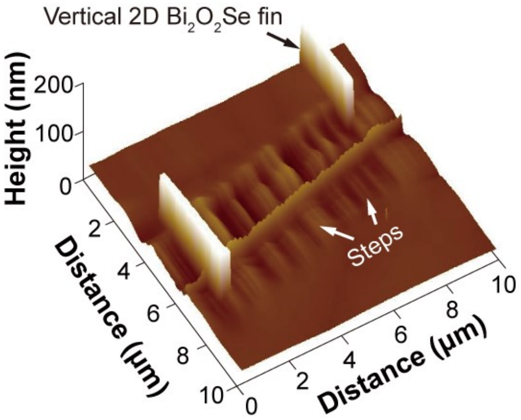

Supplementary Fig. 3. AFM image of 2D fins nucleating the step edges.

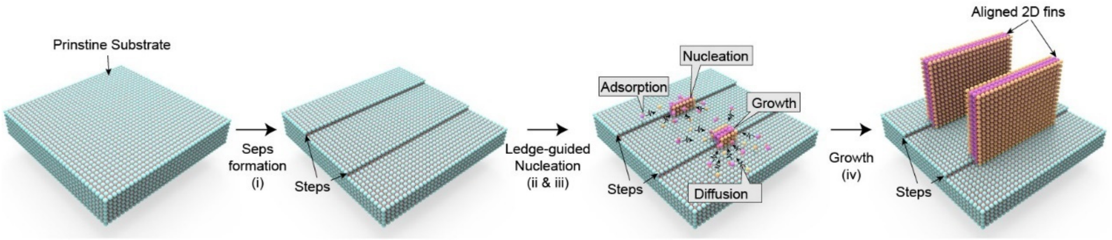

Supplementary Fig. 4. Schematic of ledge-guided epitaxy process, including steps formation, ledge-guided nucleation and growth.

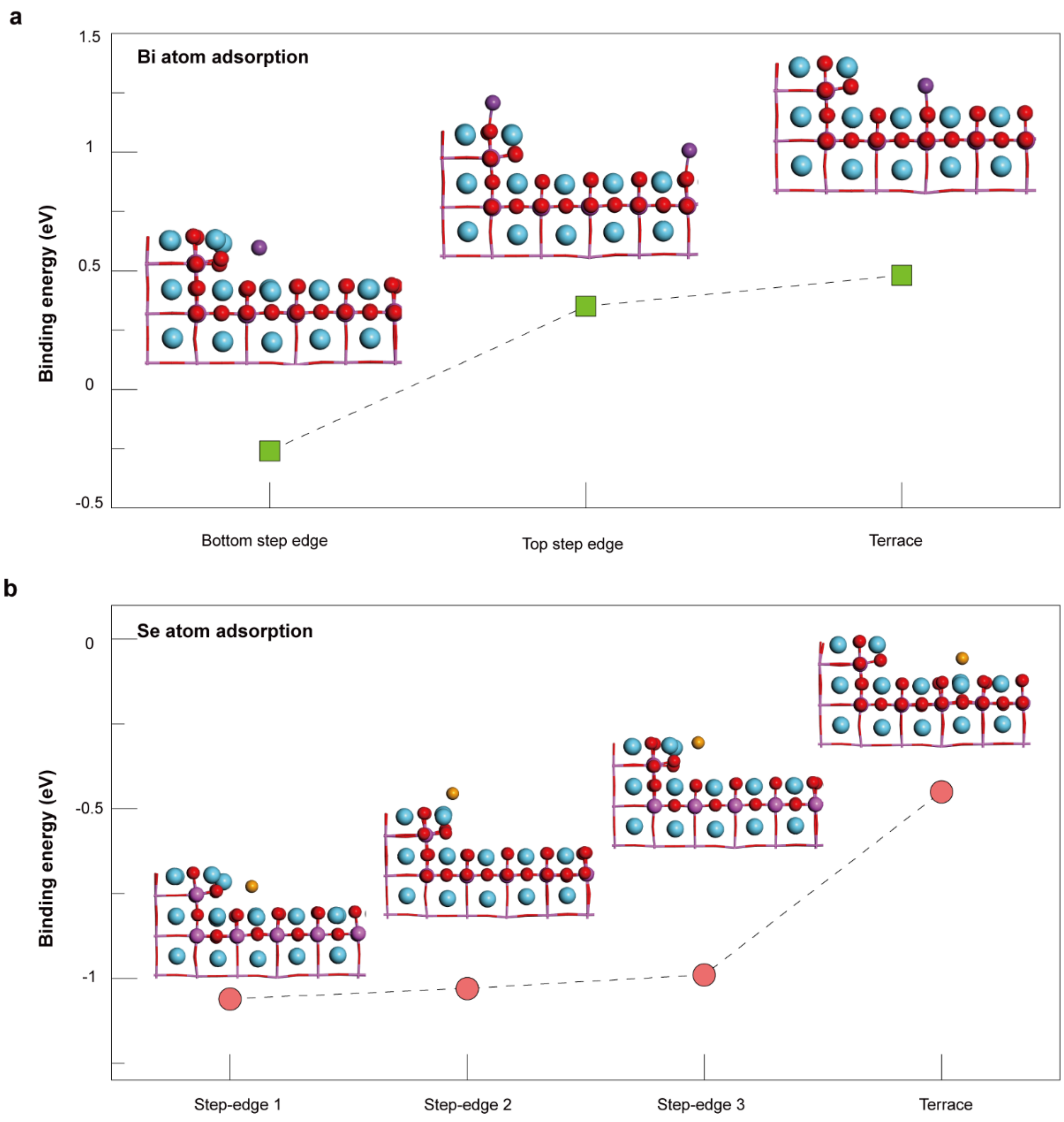

Supplementary Fig. 5. The configurations of Bi atom (a) and Se atom (b) adsorbed on the $LaAlO_3$ substrate with different sites and corresponding binding energies.

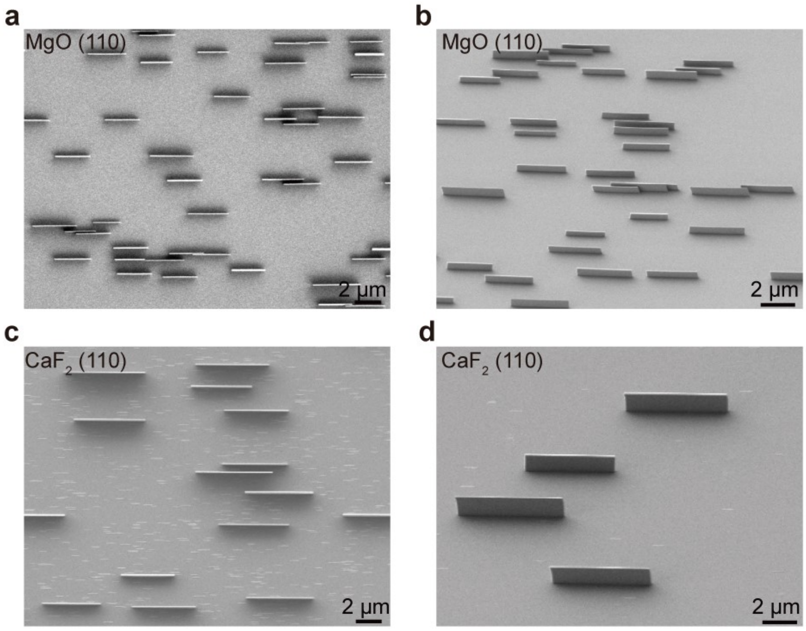

Supplementary Fig. 6. Morphology of 2D fins grown on pristine insulating substrates without steps. a, b, Top-view (**a**) and tilted-view (**b**) SEM images of 2D Bi_2O_2Se fins on MgO (110). **c, d,** Top-view (**c**) and tilted-view (**d**) SEM images of 2D Bi_2O_2Se fins on CaF_2 (110).

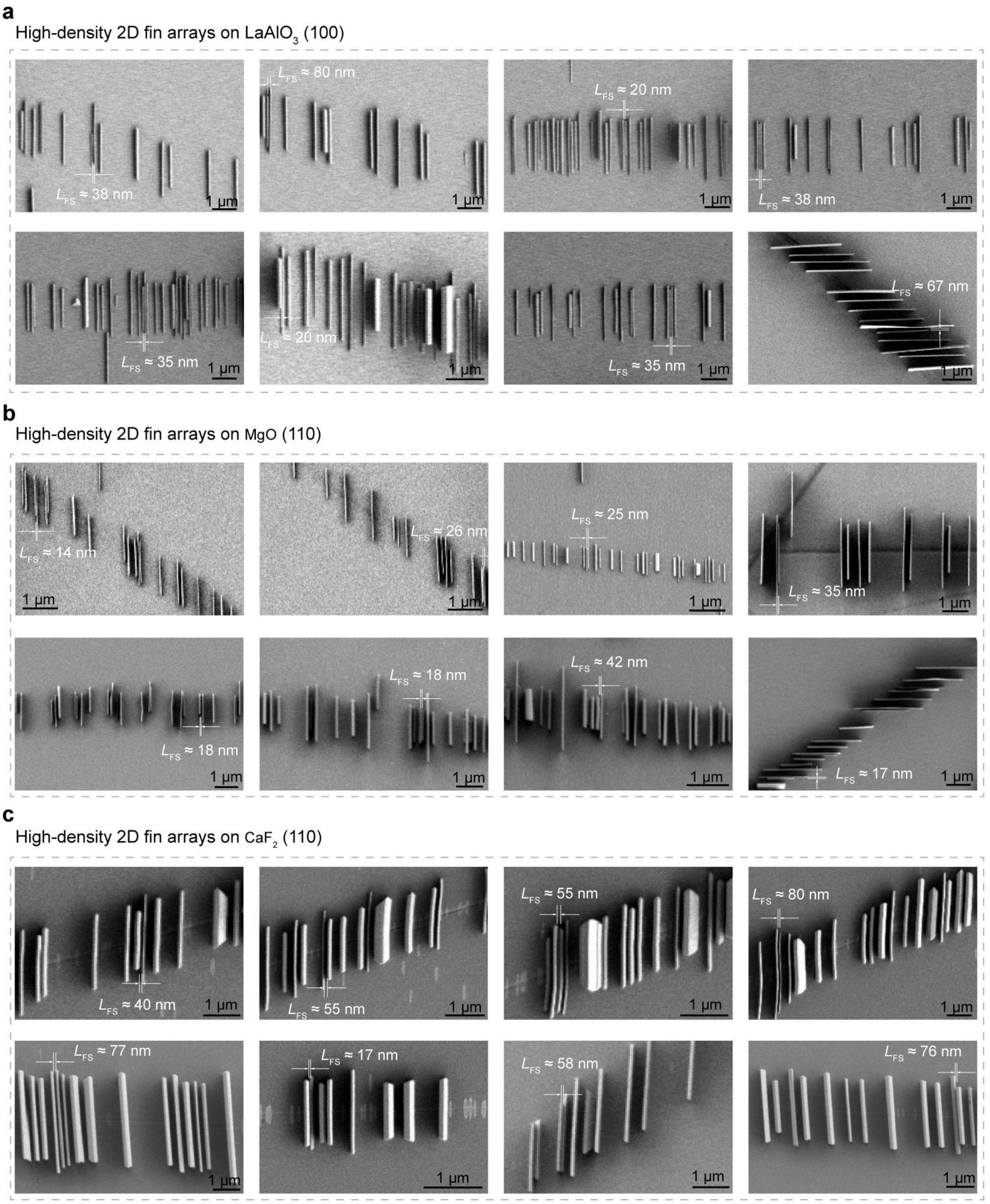

Supplementary Fig. 7. High-density 2D fin arrays on $LaAlO_3$ substrate (a), MgO substrate (b) and CaF_2 substrate (c).

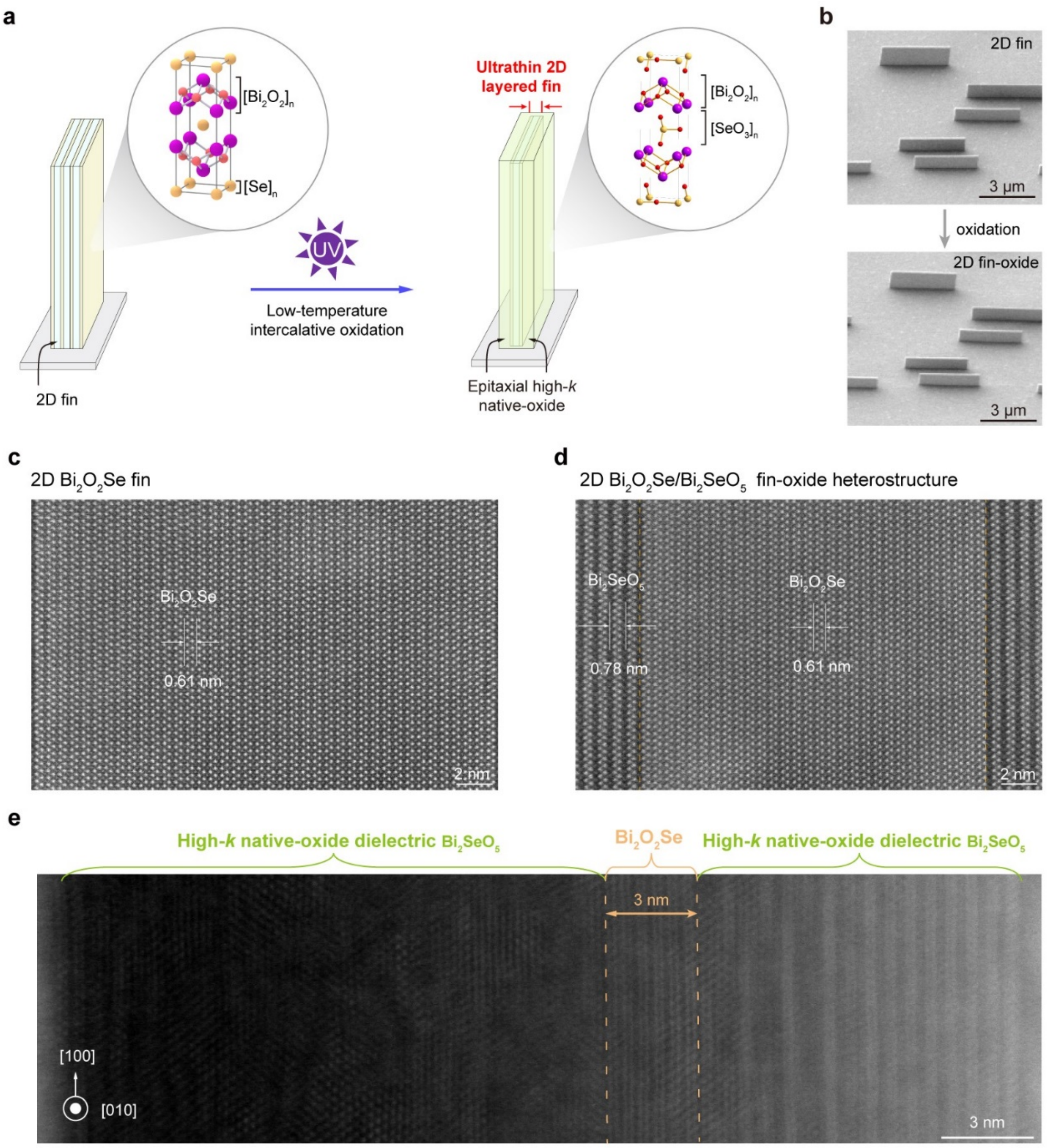

Supplementary Fig. 8. Intercalative oxidation of Bi_2O_2Se fin and structural characterization of Bi_2O_2Se/Bi_2SeO_5 fin-oxide heterostructures. a, Schematic diagram of intercalative oxidation of Bi_2O_2Se fin to Bi_2O_2Se/Bi_2SeO_5 fin-oxide heterostructure. **b,** Tilted-view SEM images of as-synthesized rectangular 2D Bi_2O_2Se fin arrays (top) and Bi_2O_2Se/Bi_2SeO_5 fin-oxide heterostructure arrays after intercalative oxidation (down) **c, d,** Cross-sectional high-resolution STEM micrograph of 2D Bi_2O_2Se fin (**b**) and Bi_2O_2Se/Bi_2SeO_5 fin-oxide heterostructure (**c**). **e,** Cross-sectional HR-STEM images of the 2D Bi_2O_2Se fin showing the ultrathin body of about 3 nm

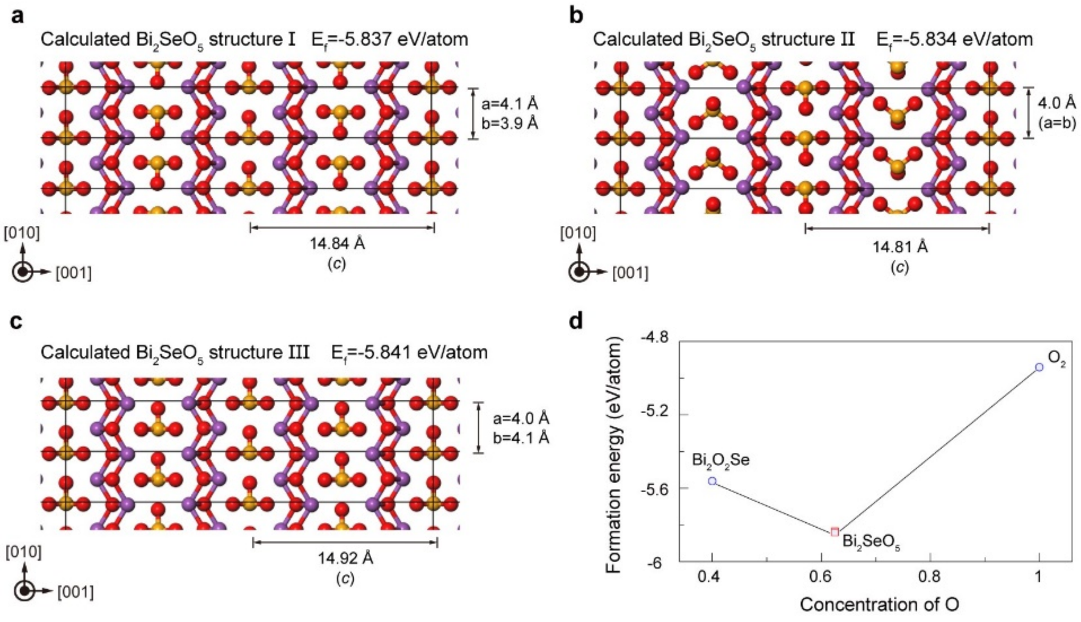

Supplementary Fig. 9. DFT calculations of formation energies and stable structures of Bi_2SeO_5. a-c, Stable crystal structures of Bi_2SeO_5 calculated by DFT. **d,** The plot of comparison of the formation energies of Bi_2O_2Se, Bi_2SeO_5 and O_2.

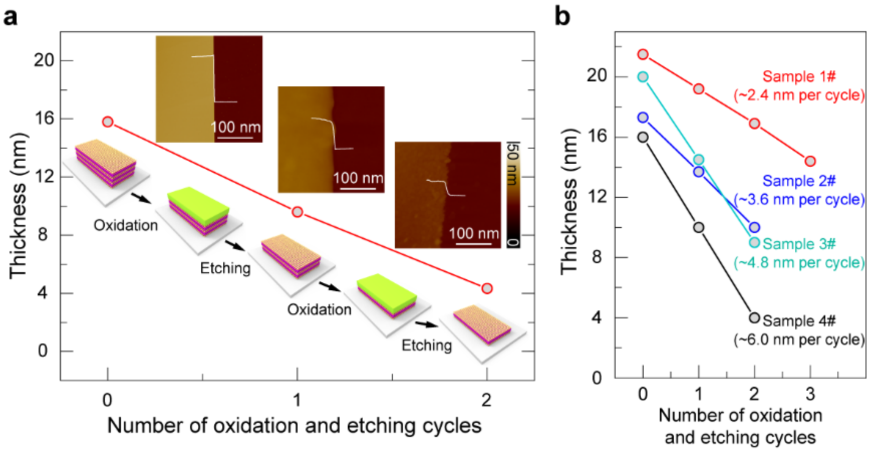

Supplementary Fig. 10. Controllable thickness scaling of 2D fins. To determine the surface roughness and thickness evolution by using AFM, the vertical fins were put down on the substrate. **a,** Thickness scaling of a Bi_2O_2Se fin in two oxidation-etching cycles. **b,** Thickness scaling of Bi_2O_2Se fins with different oxidation rate.

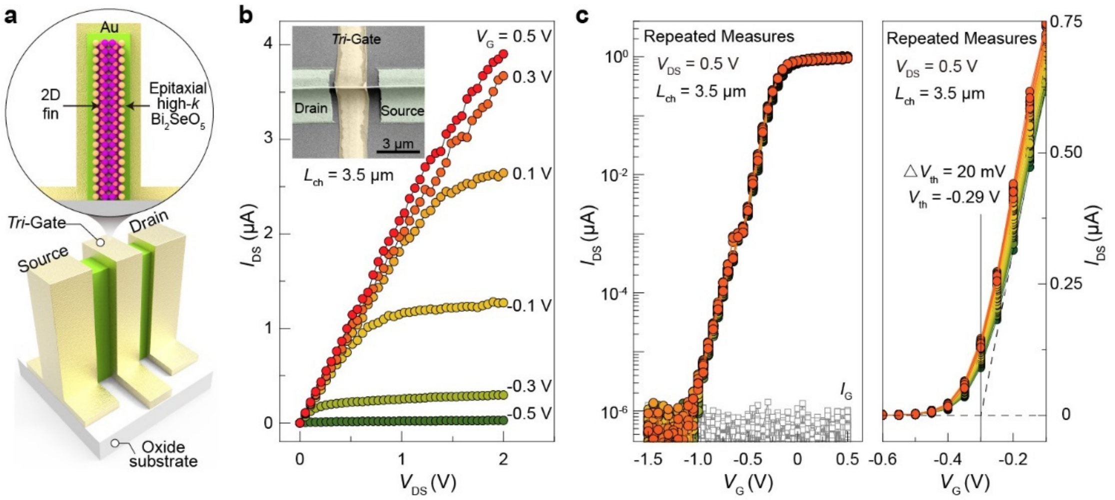

Supplementary Fig. 11. Electrical performance of 2D Bi_2O_2Se/Bi_2SeO_5 FinFET fabricated without using additional HfO_2 dielectric. a, Schematic diagram of 2D FinFET fabricated with Bi_2SeO_5 dielectric. **b, c,** Transfer (**b**) and output (**c**) curves measurement results for 50 cycles of a 2D Bi_2O_2Se/Bi_2SeO_5 FinFET with a channel length (L_{ch}) of 3.5 µm. The inset in (**b**) shows the top-view SEM image of as-fabricated 2D FinFET.

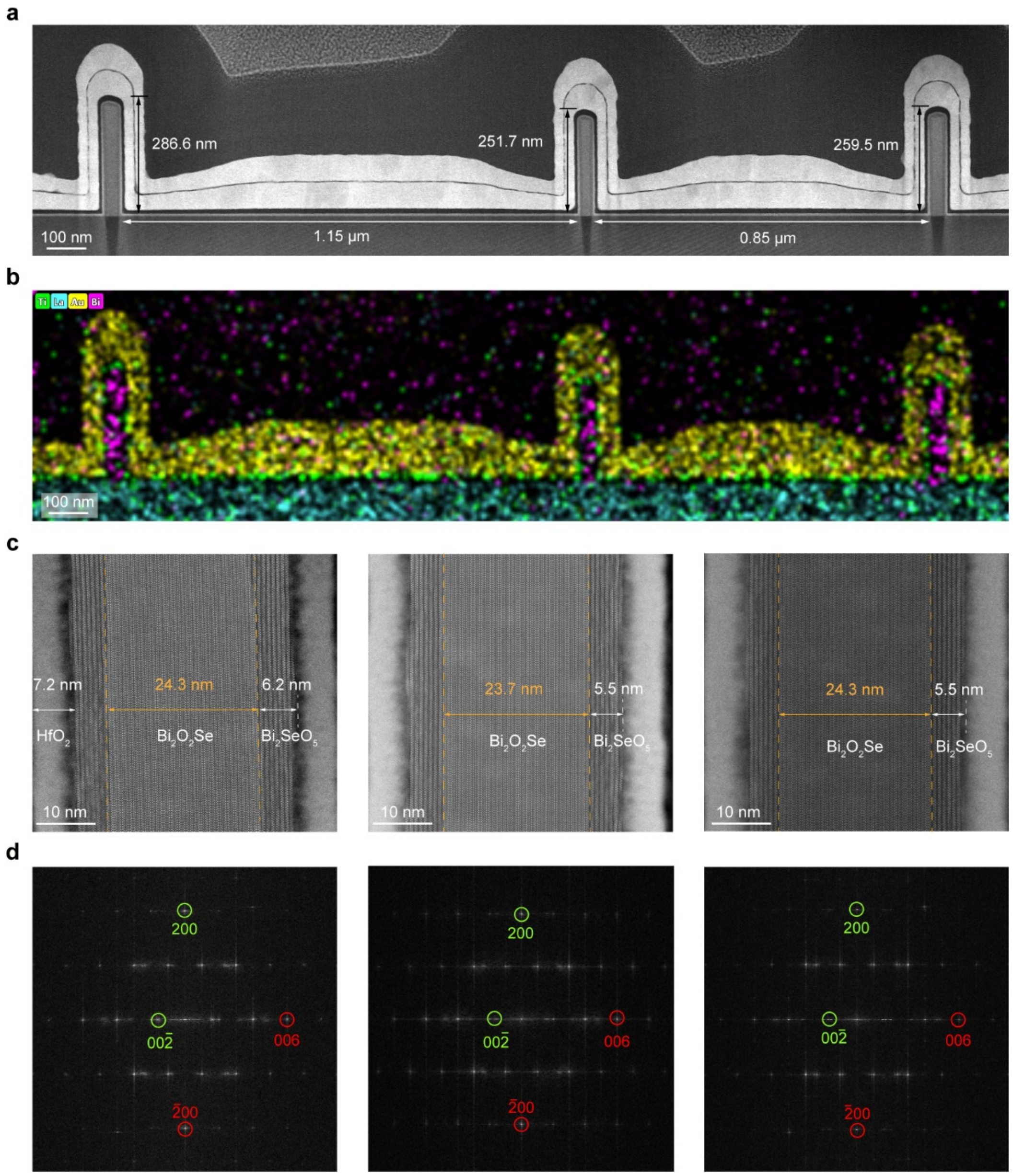

Supplementary Fig. 12. Structure characterization of a multi-fin FET with 3 fins. a, b, Cross-sectional STEM image of fin arrays (**a**) and the corresponding Energy-dispersive X-ray spectroscopy (EDS) image (**b**). **c,** The thickness measurement of different fin channels and dielectrics by high-resolution STEM images. **d,** The corresponding FFT diffraction spots of the interface in (**c**).

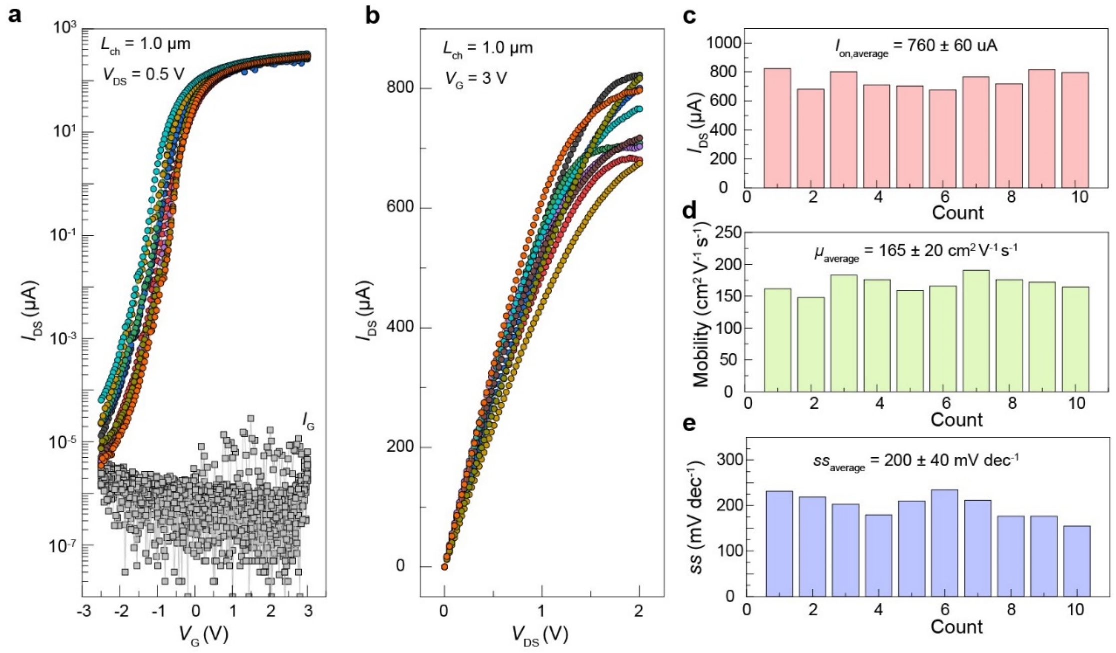

Supplementary Fig. 13. Device-to-device variability. a, b, Transfer (**a**) and output (**b**) characteristics of 10 2D multi-fin FETs (the fin number is 3). **c-e,** Statistical distributions of on-state current (**c**), mobility (**d**), and subthreshold swing (**e**) for 10 devices.

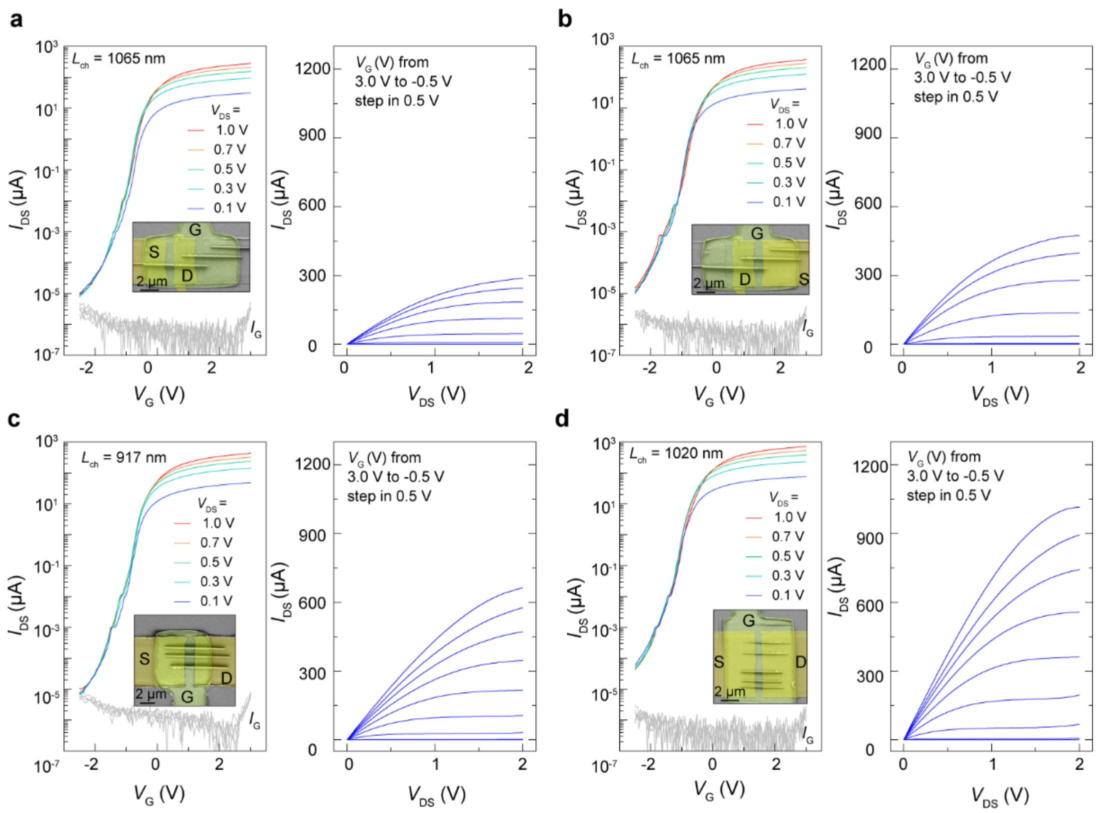

Supplementary Fig. 14. Typical output curves and transfer curves demonstrating the performance of 2D FinFETs with 1 fin (a), 2 fins (b), 3 fins (c) and 5 fins (d), respectively.

Supplementary Table 1. On-state current (I_{ON}) and transconductance (g_m) of representative 2D $Bi_2O_2Se/Bi_2SeO_5/HfO_2$ FinFETs

Device Num. (#)	*Fin Num.*	L_{Ch}/L_G^b (nm)	g_m *(uS)* (@ V_{DS} =1 V)	I_{ON} (μA) (@ V_{DS} =1 V)	$I_{ON,\ max}$ (μA) (@ V_{DS} =1 V)	Gate delay (ps)
1	1	1065	119	212	286	32.47044
2	1	959	88	200	232	30.99296
3	1	984	75	172	263	22.75557
4	2	1065	157	364	474	37.82271
5	2	822	148	325	411	32.69587
6	2	753	157	300	419	26.20741
7	2	984	135	294	366	26.62557
8	2	918	149	312	545	30.72122
9	2	780	185	308	422	26.442
10	2	1164	150	276	383	43.5104
11	2	863	155	348	463	25.89298
12	2	959	125	283	377	35.38202
13	2	1137	124	221	369	53.71785
14	3	925	201	405	532	35.77078
15	3	1096	231	560	823	37.95057
16	3	1230	203	482	681	49.48275
17	3	902	212	343	648	44.45519
18	3	1055	220	511	645	40.03384
19	3	917	210	463	487	41.05361
20	2	1274	157	395	613	41.69431
21	3	1322	177	438	663	36.01635
22	5	1020	292	707	1020	28.69273
23	2	1027	278	692	985	19.18531
